# Changing patterns of human migrations shaped the global population structure of *Mycobacterium tuberculosis* in France

**DOI:** 10.1038/s41598-018-24034-6

**Published:** 2018-04-11

**Authors:** Maxime Barbier, Oana Dumitrescu, Catherine Pichat, Gérard Carret, Anne-Sophie Ronnaux-Baron, Ghislaine Blasquez, Christine Godin-Benhaim, Sandrine Boisset, Anne Carricajo, Véronique Jacomo, Isabelle Fredenucci, Michèle Pérouse de Montclos, Charlotte Genestet, Jean-Pierre Flandrois, Florence Ader, Philip Supply, Gérard Lina, Thierry Wirth, Jean-Philippe Rasigade

**Affiliations:** 1 Institut de Systématique, Evolution, Biodiversité, UMR-CNRS 7205, Muséum National d’Histoire Naturelle, Université Pierre et Marie Curie, Ecole Pratique des Hautes Etudes, Sorbonne Universités, Paris, France; 2 Laboratoire Biologie Intégrative des Populations, Ecole Pratique des Hautes Etudes, PSL Research University, Paris, France; 3 Centre International de Recherche en Infectiologie, CIRI, University of Lyon, Lyon, France; 4 Institut des Agents Infectieux, Hospices Civils de Lyon, Lyon, France; 5 Comité Départemental d’Hygiène Sociale, CLAT69 Lyon, France; 6 Agence Régionale de Santé Auvergne-Rhône-Alpes, Lyon, France; 7 Laboratoire de Bactériologie, Institut de Biologie et de Pathologie, CHU de Grenoble, Grenoble, France; 8 Laboratoire TIMC-IMAG, UMR 5525 CNRS-UJF, UFR de Médecine, Université Grenoble Alpes, Grenoble, France; 9 Laboratoire des Agents Infectieux et d’Hygiène, CHU de Saint-Etienne, Saint-Etienne, France; 10 Laboratoire Biomnis, Lyon, France; 11 Laboratoire de Biométrie et Biologie Evolutive, UMR CNRS 5558, University of Lyon, Lyon, France; 12 Service des Maladies Infectieuses et Tropicales, Hôpital de la Croix-Rousse, Hospices Civils de Lyon, Lyon, France; 13 INSERM U1019, CNRS-UMR 8204, Center for Infection and Immunity of Lille, Institut Pasteur de Lille, Université de Lille, Lille, France

## Abstract

*Mycobacterium tuberculosis* (Mtb) exhibits a structured phylogeographic distribution worldwide linked with human migrations. We sought to infer how the interactions between distinct human populations shape the global population structure of Mtb on a regional scale. We applied the recently described timescaled haplotypic density (THD) technique on 638 minisatellite-based Mtb genotypes from French tuberculosis patients. THD with a long-term (200 y) timescale indicated that Mtb population in France had been mostly influenced by interactions with Eastern and Southern Europe and, to a lesser extent, Northern and Middle Africa, consistent with historical migrations favored by geographic proximity or commercial exchanges with former French colonies. Restricting the timescale to 20 y, THD identified a sustained influence of Northern Africa, but not Europe where tuberculosis incidence decreased sharply. Evolving interactions between human populations, thus, measurably influence the local population structure of Mtb. Relevant information on such interactions can be inferred using THD from Mtb genotypes.

## Introduction

Tuberculosis, one of the oldest diseases known to humanity, is an ongoing public health threat in many low-income countries and a re-emerging disease in several higher-income countries^1–3^. The causative agent of tuberculosis, *Mycobacterium tuberculosis* (Mtb), is thought to have co-evolved with modern humans since their expansion out of Africa ~60,000 y ago^4,5^, a situation also encountered in other bacterial pathogens such as *Helicobacter pylori*^6–8^. The dispersal of human populations was accompanied with a genetic diversification of Mtb into distinct lineages whose current distribution exhibits variable degrees of geographic specificity^9,10^. Based on this specificity, the assignment of novel Mtb strains to lineages using molecular methods reveals the possible geographic origins of the strain’s ancestry^11^. Such approaches are essential since the connection between Mtb lineages and patients origin has a practical importance for Mtb studies in low-incidence countries where a large proportion of tuberculosis cases are imported from abroad^12,13^.

More recently, phylogeographic studies of Mtb involving population genetics analysis methods have stressed the impact of social phenomena such as migrations on the evolution and population structure of Mtb lineages^10,14^. Most previous studies of Mtb phylogeography have examined continental-scale epidemics. Each country or region has a unique history of ancient and recent interactions with foreign populations that might have shaped the local Mtb population structure, but such specific events might go undetected in large-scale studies. To facilitate more local, fine-grained phylogeographic studies without the cost and time constraints of international sampling, one might consider Mtb strains infecting foreign-born patients as proxies of the circulating strains in the patients country of origin^11,15^. Under this assumption, a representative sample of Mtb isolates from an area of interest should enable to infer past transmission events involving the countries of origin of non-native patients.

Based on this rationale, we performed a comprehensive phylogeographic analysis of global Mtb strains circulating in the Rhônes-Alpes region of France, a low-incidence country where two thirds of cases involve foreign-born patients^13^. Our primary objective was to contribute an in-depth description of the influence of past interactions of French and foreign populations on the current population structure of Mtb in our region. Our secondary objective was to empirically assess the relevance of using Mtb strains from migrant patients to infer phylogeographic information on a local scale. We investigated a cohort of 638 tuberculosis patients whose infecting strains were genotyped using spoligotyping^16^ and variable number of tandem repeats (VNTR)^17^ methods. Genotype-based assignment of isolates to lineages was analyzed in combination with the country of origin of patients. To detect temporal changes in transmission patterns, we built upon a recently published phylogenetic analysis method, namely timescaled haplotypic density (THD)^18^, to estimate the intensity of Mtb exchanges between native and foreign populations over short- and long-term time scales and we interpreted the inferred results in relation with known historical and sociological events.

## Results

### Study population

**Figure 1:**
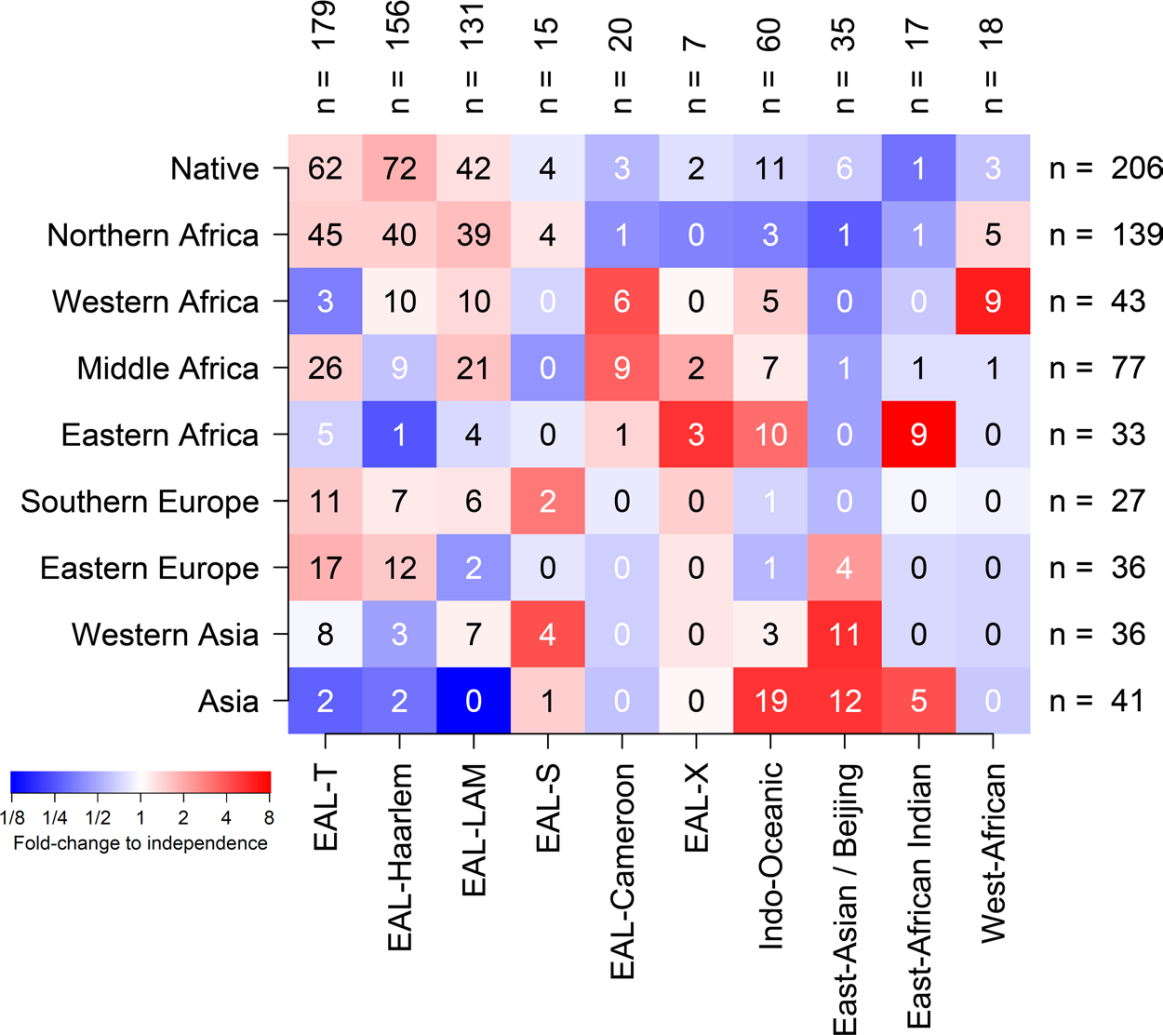
Association heatmap of major Mtb lineages and spoligotype families with patient’s region of origin. Shown are the no. of samples in each category, with row- and column-wise sample sizes indicated above and on the right of the heatmap. Colors indicate strength and direction (from blue, strongly negative, to red, strongly positive) of the association between lineage/spoligotype family and region of origin, expressed as fold-change of the observed count in each category relative to the expected count under the hypothesis of independence. Laplace smoothing was applied to proportions to avoid zero fold-changes for zero counts. Spoligotype families belonging to the Euro-American lineage are prefixed with EAL. Other lineages are designated by lineage name.

Patients were retrieved from the database of the Observatoire Rhône-Alpin des Mycobactéries (ORAM), a regional network of healthcare institutions involved in the monitoring of tuberculosis in the French Rhône-Alpes region, an area with ~6.5 million inhabitants and a population density of 150 inhabitants per km². The Rhône-Alpes region is representative of mainland France in terms of tuberculosis incidence, mean age and the proportion of foreign-born inhabitants (Supplementary Table 1). Patients were eligible for inclusion if: (1) tuberculosis was diagnosed from 2008 to 2014; (2) their country of birth was known; and (3) their infecting strain was available for genotyping using spoligotyping and mycobacterial interspersed repetitive unit (MIRU) VNTR typing with 15 loci. Patients from America and Oceania were excluded due to small sample sizes, as well as patients infected with *M. bovis*, *pinnipedii* and *microti* due to the specific transmission routes of these species (see Methods). Six hundreds and thirty-eight patients were included in the final analysis. The median age was 42 y (inter-quartile range 29–63 y), the m/f sex ratio was 1.59 and 206 patients (32.3%) were born in France. The distribution of patient geographic origins is indicated in Fig. 1.

### Phylogeographical structure of Mtb in the French population

Spoligotype-based Mtb lineages and families, inferred from the SPOLDB4 database, exhibited a highly structured phylogeographic distribution (Fig. 1). Within the Euro-American lineage, the T, Haarlem and LAM families were strongly associated with a native or North-African origin. Indo-Oceanic and East-African Indian lineages were associated with Asian or East-African origin, while the East-Asian/Beijing lineage was mostly present in Asian and West-Asian patients and, to a lesser extent, in East-European patients, consistent with previous reports of the distribution of these lineages^11,14^. Other lineages and families had more specific distributions, such as the Cameroon family found in Western- and Middle-African-born patients, and the Western-African lineage associated with a Western-African origin. Collectively, these results were in line with previous reports that migrant patients tend to be preferentially infected with endemic Mtb strains of their country of origin^11,15^.

### Timescaled haplotypic density as a measure of pathogen exchange between populations

We used THD based on MIRU haplotypes^17,19^ to quantify the intensity of Mtb exchanges between patient populations, taking migrant patients as representative of the population of their country of origin. THD is a recently described population genetics technique that analyzes the population structure of a pathogen sample to assign estimates of transmission success to individual isolates, or haplotypes^18^. THD quantifies the genetic proximity of an isolate with respect to a population with a tuning parameter, the timescale, that restricts the analysis to a given period of time before present, progressively ignoring events older than the timescale. The THD measure was shown in simulations and in an empirical study of Mtb to reflect the number of common ancestors of the isolate and the sampled population in the timescale, which in turn reflects the density of transmission events in the ancestry of the isolate, that is, the isolate’s transmission success.

In its original application^18^, THD assigns a measure of success to an isolate based on its similarity to other isolates in the whole dataset. If isolates are clustered into groups and THD similarity is measured from isolates in a group relative to those in another group (rather than to the whole dataset), then this similarity reflects the intensity of isolate exchanges between the groups over the considered timescale. Based on this rationale, we generalized the use of THD to groups of isolates and we called the resulting measure cross-THD. Here, isolates were grouped according to patient’s geographic origin, allowing to perform fine-grained phylogeographic analyses focused on specific timescales. To ease comparisons between groups, average cross-THDs were normalized to sum to unity (see Methods). The cross-THD score of a group relative to itself, termed self-THD, reflects the intensity of exchanges within the group relative to exchanges with other groups. Self-THD, thus, reflects the group’s isolation relative to other groups.

**Figure 2:**
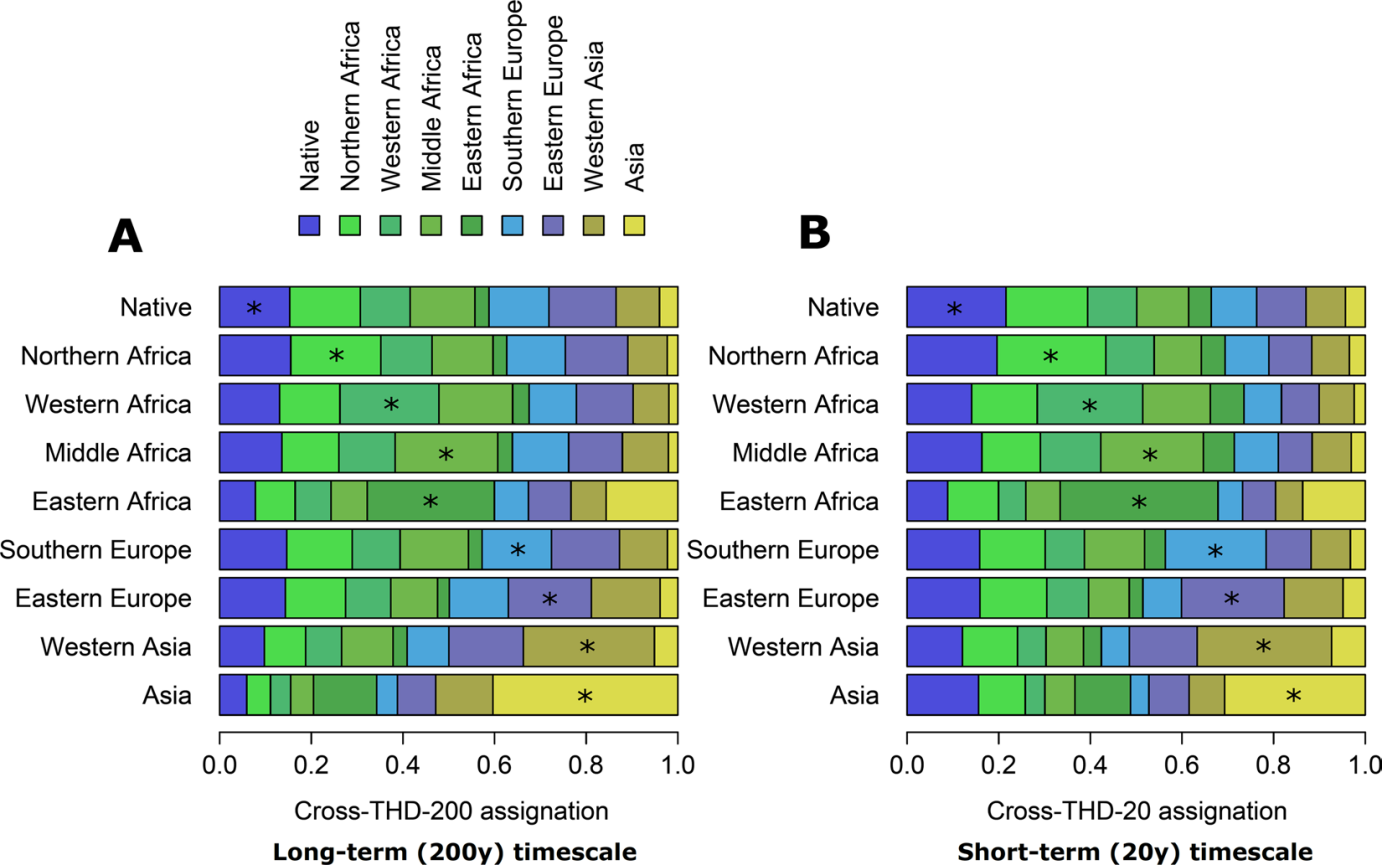
Phylogeographic structure of Mtb strains from the Rhône-Alpes region of France. Cross-THDs with timescales of 200 y (**A**) and 20 y (**B**) were computed based on 15-loci MIRU-VNTR haplotypes in 638 isolates. In each row, cross-THDs of all geographical groups are shown relative to the reference group indicated on the left. Cross-THDs estimate the relative intensity of Mtb exchanges between the reference and the other groups over the indicated timescale. An asterisk indicates self-THD, which estimates the relative intensity of exchanges within the reference group of the row.

We examined exchanges of Mtb strains between populations in our cohort using 20 y (short-term) and 200 y (long-term) THD timescales, consistent with our previous work on Mtb MIRU haplotypes^18^. For each timescale, cross- and self-THDs were computed across all regions of origin including France (native patients). This analysis revealed several important phylogeographic characteristics in our cohort (Fig. 2). First, self-THD scores (identified with asterisks in the figure) were below 50%, suggesting that Mtb population structure in all regions has been substantially influenced by exchanges with other regions. Second, self-THD scores over the 200 y timescale were comparably low in patients born in Europe and non-Eastern Africa, with mean scores ranging from 15.3% for France to 22.4% for Middle Africa. The higher self-THD scores for Eastern Africa, Western Asia and Asia (27.8, 28.6 and 40.4%, respectively; all differences had P < 0.05; see Supplementary Fig. 1) suggested that Mtb population structures in these regions have been less influenced by exchanges with other regions. Third, this pattern of self-THD differences was still present when using the 20 y timescale (Fig. 2B and Supplementary Fig. 1D), however the amplitude of the differences markedly decreased. We then used cross-THD analyses in non-native patient isolates to examine whether the time spent in France influenced the probability of infection with an isolate of the French endemic background. This analysis was restricted to the 148 non-native patients with known date of arrival in France. The distributions of age and delay from arrival in France to tuberculosis diagnosis are summarized in the Supplementary Table 2. In isolates infecting non-native patients, the cross-THD relative to native patient isolates was computed as a proxy of the strain relatedness to French-endemic strains. Cross-THD did not correlate with the time spent in France, using either a 200 y timescale (rho = 0.00; P = 0.99) or a 20 y timescale (rho = 0.02, P = 0.79). Cross-THD was not significantly correlated with patient age using a 200y timescale (rho = 0.09, P = 0.064) but weakly correlated using a 20 y timescale (rho = 0.11, P = 0.020). We suspected that this correlation was driven by patients of Northern African origin, who were older than other non-native patients and whose community has an older history of contacts with French natives. Indeed, the correlation vanished (P = 0.96) after excluding patients of Northern African origin. Collectively, these results suggest that cross-THD relative to native patients does not evolve through time in non-native patients.

### Patterns of Mtb exchanges with the French population changed over time

**Figure 3:**
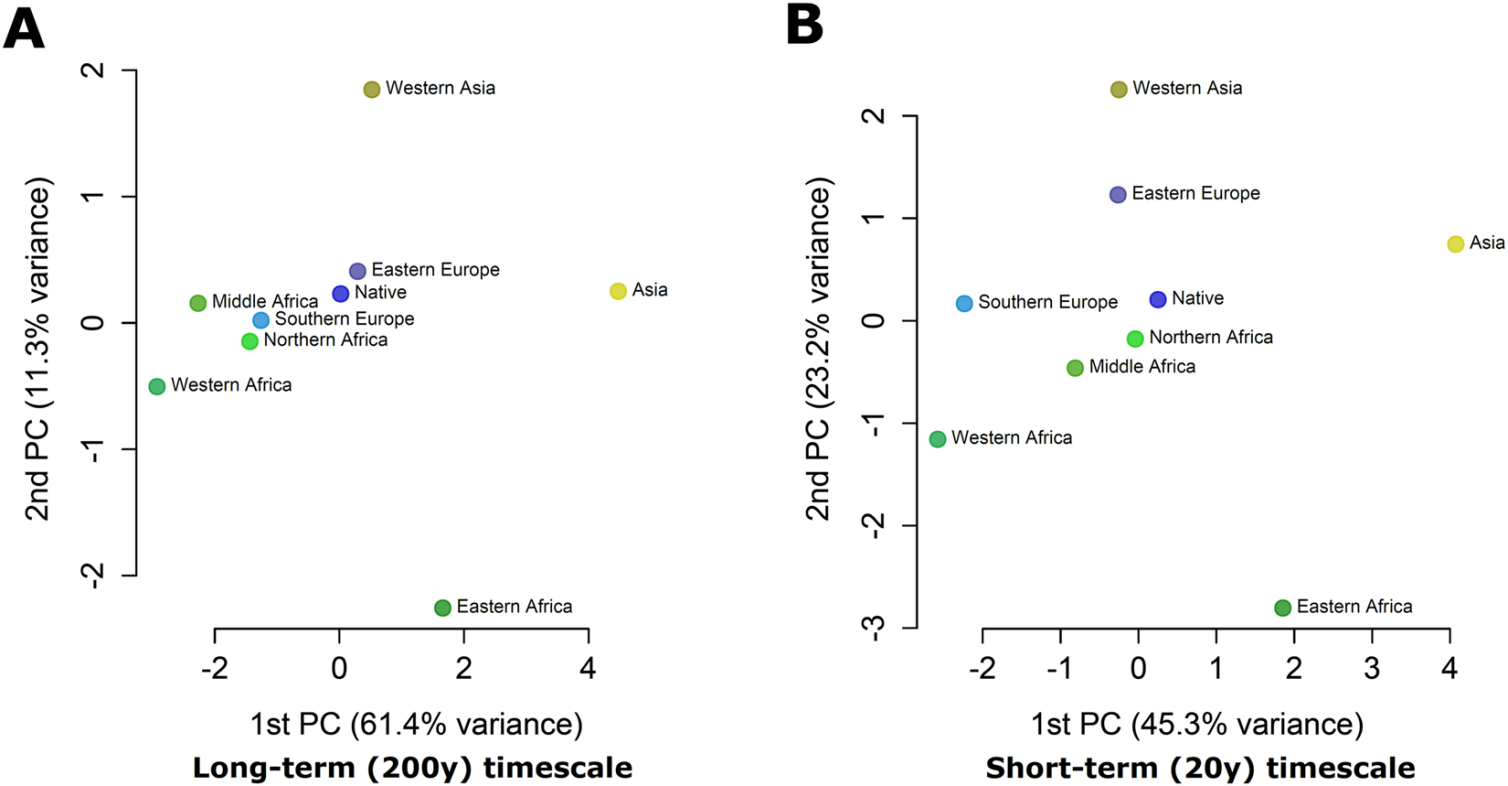
THD-based similarity between Mtb population structures using long-term (**A**) and short-term (**B**) timescales. Phylogenetic proximities between regions of origin were estimated by transforming pairwise cross-THDs into distances visualized using multidimensional scaling. PC, principal component.

To visualize relationships between the population structures of all geographic regions, cross-THDs were transformed into dissimilarity measures (see Methods) and regions were projected as points on a plane by means of metric multidimensional scaling (MDS; Fig. 3). Using both the 20 y and 200 y timescales, the French-native group was projected onto the most central position on the first MDS plane, which suggested that this group exhibited on average the largest similarity with all other groups, consistent with its low self-THD score. MDS projection highlighted strong similarities of Mtb population structure between France, Eastern and Southern Europe over the 200 y timescale, with a gradient of similarity consistent with the geographical disposition of the contributing regions, from Western and Middle Africa to Northern Africa, Southern Europe and France, while Eastern Africa, Western Asia and Asia were projected further away from the French-native group. After focusing the analysis to the more recent past by using a 20 y timescale, several changes occurred in the MDS projection: although the gradient between non-Eastern African regions remained, Northern Africa became the closest region to France, while Southern and Eastern Europe were projected away from France, suggesting changes in the intensity of Mtb exchanges between these regions compared to the long-term timescale.

**Figure 4:**
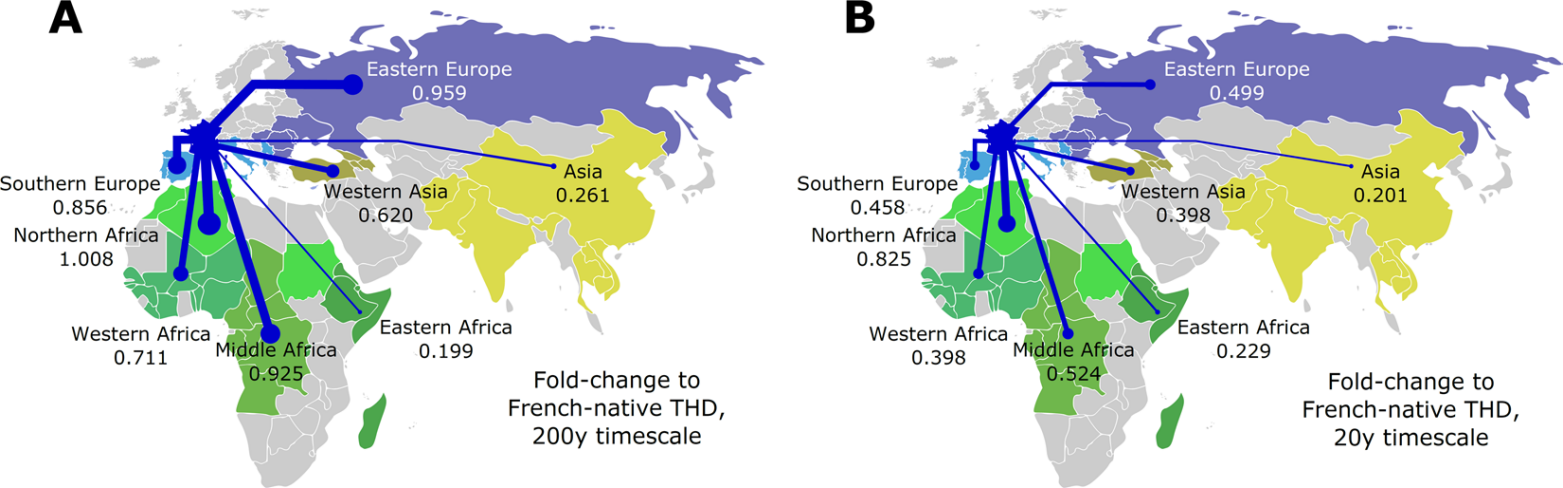
Long- and short-term contributions of interactions between France and other regions to the local Mtb population structure. Line widths are proportional to fold-change of cross-THDs relative to self-THD in French-native patients, using timescales of 200 y and 20 y (**A** and **B**, respectively). Maps were prepared with Inkscape v0.91 software (https://inkscape.org) and adapted from public domain vectorized map file accessible at: https://commons.wikimedia.org/wiki/File:BlankMap-World6.svg.

The MDS projections took into account all pairwise similarities between regions without focusing only on similarities with the French-native group. To specifically examine the contribution of each region to the population structure of strains infecting French-native patients, ignoring other pairwise similarities, cross-THDs of each region relative to the French native group were expressed as fold-changes to the self-THD of the native group and displayed on a map (Fig. 4). This analysis confirmed the major contribution of exchanges with European and non-Eastern African populations to the long-term population structure of Mtb in French-native patients, as well as the relative decline of the contribution of most regions excepted North Africa when narrowing the THD focus to the short-term 20 y timescale.

## Discussion

In this analysis of 638 tuberculosis patients from a low-incidence area, we identified distinct patterns of international Mtb transmission that evolved with time and that highlighted how the history of a local human population contributes to shape its Mtb population structure. Our results also demonstrated how the THD technique, which was developed initially to estimate the transmission success of single isolates^18^, can be applied to phylogeographic analyses in a straightforward fashion.

Comparisons of long- and short-term cross-THDs in Mtb strains of French-native and non-native patients unraveled patterns of genetic similarities suggesting how past and recent contacts of the French population with regions of varying Mtb prevalences have influenced the current Mtb population structure. Regions that contributed the most to this structure over a 200 y timeframe were either geographically close to France, such as Southern and Eastern Europe, or included former French colonies, such as Northern, Middle and Western Africa (Fig. 3). These inferences are consistent with historical records, documenting that tuberculosis transmission between inhabitants of mainland France and African territories increased rapidly in the first half of the 20^th^ century, favored by economic exchanges and the transportation of military recruits, especially during the course of the First World War^20^.

Shortening the THD timescale to 20 y, the similarities in these European and African regions with French Mtb were nearly halved (e.g., from 0.96 to 0.50 and from 0.86 to to 0.458 for Eastern and Southern Europe, respectively; Fig. 4) with the notable exception of Northern Africa. These patterns correlate with known changes in both the intensity of contacts with mainland France and in the regional prevalence of Mtb. In Middle and Western Africa, Mtb prevalence has remained high^3^, however the intensity of contacts with the French-native population has decreased sharply after the decolonization movement that followed the Second World War. An opposite situation could prevail for Southern and Eastern Europe; these regions remain the major source of immigration to France^21^, however they benefited from a sharp decrease in Mtb prevalence in the long term which has probably contributed to lower the transmission. Finally, contacts of French inhabitants with Northern African countries such as Algeria, Morocco and Tunisia have remained very frequent^21,22^ and Mtb prevalence in these countries is still high^23^, which might explain why the cross-THD contribution of Northern Africa did not decrease substantially from the 200 y to the 20 y timeframe.

Importantly, our analyses relied on the assumption that migrant patients could be taken as representatives of the population of their country of origin with respect to Mtb molecular epidemiology. This assumption is violated when migrant patients become infected during travel or once in France or when native patients become infected when abroad. Patients born outside Europe and Northern Africa exhibited an Mtb population structure that was clearly distinct from that of native patients (Fig. 1). Moreover, cross-THD of non-native patient isolates relative to native patient isolates did not correlate with the time spent in France. This suggested that infection by isolates of the French endemic background was rare in the migrant population, as was expected given the lower tuberculosis prevalence compared to Asia and Africa. The proximity, however, between population structures found in patients born in France and Northern Africa might have been enhanced by two sociological peculiarities. First, 2^nd^ or 3^rd^-generation immigrants frequently visit family in their country of origin^24^, increasing the odds of contact with Mtb strains endemic in Northern Africa. Second, migration waves from Northern Africa began before those from Asia and sub-Saharan Africa. This situation has probably contributed to blur the distinction between native and Northern African patients. It is unlikely, however, to have biased our conclusion regarding the major contribution of contacts with Northern Africa to the population structure of Mtb in France.

To conclude, we show that tuberculosis genotyping data obtained from a routine surveillance programme convey enough phylogenetic information to extract meaningful inferences regarding past epidemiological events. Our approach, based on the easily implemented THD technique, allowed to consider Mtb as a whole rather than to focus on a single lineage, as was the case with most recent phylogeographical studies of tuberculosis. The application of similar approaches to international settings such as Europe, which combines a low tuberculosis prevalence and intense migration flows, might provide important information relevant to the current re-emergence of tuberculosis.

## Methods

### Ethics statement

This retrospective, cross-sectional, observational multicentric study was approved by the Comité de Protection des Personnes Sud-Est IV under no. DC-2011-1306. The sociodemographic data collected along with Mtb isolates included sex, age, country of origin and the date of arrival in France of non-native patients. Written consent of participants was not obtained, in accordance with French regulations, due to anymous treatment of data and the non-interventional nature of the study.

### Patient population and collection of data

The 2008–2014 tuberculosis patient cohort of the ORAM was described previously and detailed data were made publicly available^18^. Briefly, 1,746 patients with available *M. tuberculosis* complex strains were identified. Countries of birth were coded according to ISO3166 standard (http://www.iso.org/iso/home/standards/country_codes.htm) and assigned to world regions according to the United Nations Geoscheme (http://millenniumindicators.un.org/unsd/methods/m49/m49regin.htm) with the exception of Central, Eastern, Southern and Southeastern Asia UN regions (n = 1, 4, 9 and 28 patients, respectively), which were pooled into a single Asia region to avoid small per-region sample sizes. Of note, we also complied with the UN Geoscheme convention that the Russian Federation, despite its territory spanning both Europe and Asia, was assigned to the Eastern Europe world region. Exclusion criteria were: (1), ambiguous MIRU-VNTR profile (i.e., undefined number of repeats at any of the 15 loci; n = 105); (2), unknown country of origin (n = 958); (3), birth outside Europe, Africa and Asia due to small sample size (n = 6); and (4), infection with species other than Mtb whose transmission does not primarily involve interhuman contact (n = 39), including *M. bovis*, *pinnipedii* and *microti*, to avoid interpretation bias.

### Isolate genotyping and family/lineage assignment

Spoligotyping was performed as described elsewhere^16^. Spoligotypes were compared to those of the SpolDB4 database^25^ to assign isolates to families including AFRI, Beijing, Cameroon, CAS, Haarlem, LAM, S, T and X^26^, which were then reclassified into 6 major genome sequence- (or genomic deletion-) based lineages, including e.g. the East-African Indian (lineage 3), East Asian (lineage 2), Euro-American (lineage 4), Indo-Oceanic (lineage 1) and West African (lineages 5 and 6) lineages^27^, according to known correspondences^28^.

### Timescaled haplotypic density

THD was implemented for the R platform (The R Foundation for Statistical Computing, Vienna, Austria) using publicly available software code^18^. In the THD framework, the genetic distances between isolates serve as a basis to assign a measure of density to each isolate. The density function is estimated using kernel density estimation with a geometric kernel, where the kernel bandwidth parameter is expressed in units of time (here, the timescale) based on a functional relationship between genetic distance and the time to the most recent common ancestor (TMRCA) under the infinitely many alleles model^29^. In its original implementation, THD was measured for a given haplotype relative to all other haplotypes in a population. THD was interpreted as a proxy of the number of common ancestors during the considered timescale, a quantity related to the transmission success of the pathogen haplotype. In the present study, THD was measured relative to haplotypes in other groups to reflect the intensity of transmission events between groups during the timescale (rather than within a single group). Cross-THDs between groups based on geographic regions were reported as the means of individual THDs in each group, normalized to add to 1. Cross-THD from a group relative to itself was referred to as self-THD. THD parameters, namely timescales and mutation rate, were set as described in our previous study of MIRU-VNTR data^18^. Source code of THD and cross-THD computation routines, along with an example of cross-THD analysis using R software, are provided in the Supplementary Information files.

### Multidimensional scaling

Cross-THDs were transformed into dissimilarity (distance) measures to ease visualization by means of multidimensional scaling^30^. The transformation involved: (1) adding the asymmetric matrix of inverse pairwise cross-THDs to its transposed matrix to obtain a symmetric matrix; (2) obtaining a matrix with unit diagonal elements by dividing each element with the outer product of the square roots of the diagonal entries, similar to the transformation of a covariance matrix into a correlation matrix; and (3) subtracting 1 to all elements of the normalized matrix so that the distance from one point to itself is zero. It is easily verified that the elements of the resulting matrix fulfill the three conditions of a distance measure (formally, a semimetric), namely non-negativity, symmetry and identity of the indiscernibles. Finally, the THD-based dissimilarity matrix was taken as input to the multidimensional scaling procedure, which projected each group as a point on a plane such that inter-group distances were the best linear approximations of THD-based dissimilarity.

## Electronic supplementary material

Supplementary Information

Supplementary Source Code

Dataset 1

## References

[1] Couvin D, Rastogi N (2015). Tuberculosis - A global emergency: Tools and methods to monitor, understand, and control the epidemic with specific example of the Beijing lineage. Tuberculosis (Edinb).

[2] The Lancet Respiratory Medicine (2015). Changing minds about tuberculosis. Lancet Respir Med.

[3] WHO|Global tuberculosis report 2015. *WHO* Available at: http://www.who.int/tb/publications/global_report/en/. (Accessed: 23rd February 2016).

[4] Comas I (2013). Out-of-Africa migration and Neolithic coexpansion of *Mycobacterium tuberculosis* with modern humans. Nat. Genet..

[5] Barbier, M. & Wirth, T. The Evolutionary History, Demography, and Spread of the *Mycobacterium tuberculosis* Complex. *Microbiol Spectr* **4** (2016). 10.1128/microbiolspec.TBTB2-0008-2016 27726798

[6] Wirth T, Meyer A, Achtman M (2005). Deciphering host migrations and origins by means of their microbes. Mol. Ecol..

[7] Linz B (2007). An African origin for the intimate association between humans and *Helicobacter pylori*. Nature.

[8] Moodley Y (2012). Age of the association between *Helicobacter pylori* and man. PLoS Pathog..

[9] Wirth T (2008). Origin, spread and demography of the *Mycobacterium tuberculosis* complex. PLoS Pathog..

[10] Stucki D (2016). *Mycobacterium tuberculosis* lineage 4 comprises globally distributed and geographically restricted sublineages. Nat. Genet..

[11] Reed MB (2009). Major *Mycobacterium tuberculosis* lineages associate with patient country of origin. J. Clin. Microbiol..

[12] Dale JW (2005). Origins and properties of *Mycobacterium tuberculosis* isolates in London. J. Med. Microbiol..

[13] Pichat C (2016). Combined Genotypic, Phylogenetic, and Epidemiologic Analyses of *Mycobacterium tuberculosis* Genetic Diversity in the Rhône Alpes Region, France. PLoS ONE.

[14] Merker M (2015). Evolutionary history and global spread of the *Mycobacterium tuberculosis* Beijing lineage. Nat. Genet..

[15] Fallico L (2014). Four year longitudinal study of *Mycobacterium tuberculosis* complex isolates in a region of North-Eastern Italy. Infect. Genet. Evol..

[16] Kamerbeek J (1997). Simultaneous detection and strain differentiation of *Mycobacterium tuberculosis* for diagnosis and epidemiology. J. Clin. Microbiol..

[17] Supply P (2006). Proposal for standardization of optimized mycobacterial interspersed repetitive unit-variable-number tandem repeat typing of *Mycobacterium tuberculosis*. J. Clin. Microbiol..

[18] Rasigade J-P (2017). Strain-specific estimation of epidemic success provides insights into the transmission dynamics of tuberculosis. Sci Rep.

[19] Supply P (2001). Automated high-throughput genotyping for study of global epidemiology of *Mycobacterium tuberculosis* based on mycobacterial interspersed repetitive units. J. Clin. Microbiol..

[20] Eckart, W. U. *Man, Medicine, and the State: The Human Body as an Object of Government Sponsored Medical Research in the 20th Century* (Franz Steiner Verlag, 2006).

[21] Institut National de la Statistique et des Etudes Economiques. Répartition des étrangers par nationalité en 2014. (2014). Available at: https://www.insee.fr/fr/statistiques/2381750. (Accessed: 13th July 2017)

[22] D’Albis H, Boubtane E (2015). Caractérisation des flux migratoires en France à partir des statistiques de délivrance de titres de séjour (1998-2013). Population.

[23] Khyatti M (2014). Infectious diseases in North Africa and North African immigrants to Europe. Eur. J. Public Health.

[24] Armand L (2007). Les touristes français à l’étranger en 2006: résultats issus du suivi de la demande touristique (Direction du Tourisme). Bulletin Epidémiologique Hebdomadaire..

[25] Brudey K (2006). *Mycobacterium tuberculosis* complex genetic diversity: mining the fourth international spoligotyping database (SpolDB4) for classification, population genetics and epidemiology. BMC Microbiol..

[26] Comas I, Homolka S, Niemann S, Gagneux S (2009). Genotyping of genetically monomorphic bacteria: DNA sequencing in *Mycobacterium tuberculosis* highlights the limitations of current methodologies. PLoS ONE.

[27] Gagneux S (2006). Variable host-pathogen compatibility in *Mycobacterium tuberculosis*. Proc. Natl. Acad. Sci. USA.

[28] Shabbeer A (2012). TB-Lineage: an online tool for classification and analysis of strains of *Mycobacterium tuberculosis* complex. Infect. Genet. Evol..

[29] Walsh B (2001). Estimating the time to the most recent common ancestor for the Y chromosome or mitochondrial DNA for a pair of individuals. Genetics.

[30] Izenman, A. J. *Modern Multivariate Statistical Techniques: Regression, Classification, and Manifold Learning*. (Springer Publishing Company, Incorporated, 2008).

