# Supplementary material for: Changing patterns of human migrations shaped the global population structure of Mycobacterium tuberculosis in France
Source: Sci Rep. 2018 Apr 11;8:5855. doi: 10.1038/s41598-018-24034-6 (PMC5895845; doi:10.1038/s41598-018-24034-6)
Supplement: Supplementary file 1 — Supplementary Information [file 41598_2018_24034_MOESM1_ESM.pdf]

Changing patterns of human migrations shaped the global population structure of *Mycobacterium tuberculosis* in France

Maxime Barbier, Oana Dumitrescu, Catherine Pichat, Gérard Carret, Anne-Sophie Ronnaux-Baron, Ghislaine Blasquez, Christine Godin-Benhaim, Sandrine Boisset, Anne Carricajo, Véronique Jacomo, Isabelle Fredenucci, Michèle Pérouse de Montclos, Charlotte Genestet, Jean-Pierre Flandrois, Florence Ader, Philip Supply, Gérard Lina, Thierry Wirth & Jean-Philippe Rasigade

Supplementary Information File

Contents:

Supplementary Table 1. Population characteristics in mainland France and the Rhône-Alpes region.

Supplementary Table 2. Age and delay from arrival in France to tuberculosis diagnosis in non-native patients.

Supplementary Figure 1. Distribution of self-THDs in isolates from French-native and non-native patients with tuberculosis.

Supplementary Methods 1. Practical implementation of THD and cross-THD analyses.

Supplementary Table 1. Population characteristics in mainland France and the Rhône-Alpes region.

	Rhône-Alpes region	Mainland France
Tuberculosis incidence per 100,000 inhabitants per year	7.1	8.1
Mean age	40	41
Immigrants (%)	9.1	9.1

Sources, accessed 2018-02-21

http://invs.santepubliquefrance.fr/beh/2017/7/2017_7_1.html

https://www.insee.fr/fr/statistiques/1893198

https://www.insee.fr/fr/statistiques/zones/2874056?geo=FRANCE-1+REG-84&debut=0

Supplementary Table 2. Age and delay from arrival in France to tuberculosis diagnosis in non-native patients.

Region of origin	No. of patients	Age[a]	No. with known date of arrival in France (%)	Delay from arrival to tuberculosis diagnosis[a]
Northern Africa	139	55.0 [34.5 - 70.5]	34 (24.46)	13.0 [3.0 - 32.0]
Western Africa	43	30.0 [24.5 - 36.5]	21 (48.84)	4.0 [1.0 - 11.0]
Middle Africa	77	29.0 [22.0 - 40.0]	28 (36.36)	3.0 [1.0 - 6.5]
Eastern Africa	33	31.0 [27.0 - 38.0]	16 (48.48)	4.5 [2.2 - 8.0]
Southern Europe	27	48.0 [33.0 - 76.5]	7 (25.93)	3.0 [2.0 - 8.5]
Eastern Europe	36	38.0 [27.2 - 47.2]	9 (25.00)	0.0 [0.0 - 0.0]
Western Asia	35	41.0 [31.0 - 51.0]	14 (40.00)	5.0 [1.0 - 14.2]
Asia	41	44.0 [31.0 - 54.0]	19 (46.34)	15.0 [2.5 - 29.5]

[a]Median and interquartile range

A

	Native	Southern Europe	Eastern Europe	Northern Africa	Western Africa	Middle Africa	Eastern Africa	Western Asia
Southern Europe	0.948861							
Eastern Europe	0.224679	0.36109						
Northern Africa	2.17E-03	0.095622	0.527155					
Western Africa	0.003525	0.040451	0.229862	0.38034				
Middle Africa	4E-05	0.011674	0.099573	0.131226	0.746771			
Eastern Africa	3.38E-07	0.000174	0.002017	0.001224	0.040224	0.046917		
Western Asia	1.61E-08	4.42E-05	0.000586	0.000215	0.016535	0.0174	0.780512	
Asia	2.38E-27	1.30E-14	1.56E-13	2.01E-18	6.22E-11	1.79E-12	3.39E-05	7.64E-05

B

	Native	Southern Europe	Eastern Europe	Northern Africa	Western Africa	Middle Africa	Eastern Africa	Western Asia
Southern Europe	0.900156							
Eastern Europe	0.773476	0.917668						
Northern Africa	0.194166	0.578265	0.62803					
Western Africa	0.574611	0.780474	0.852099	0.781087				
Middle Africa	0.683484	0.897602	0.990255	0.534994	0.835007			
Eastern Africa	6.98E-06	0.001566	0.000984	0.000282	0.001152	0.000145		
Western Asia	0.004887	0.057479	0.05235	0.049813	0.06599	0.024343	0.159214	
Asia	4.93E-04	0.02104	0.016926	0.010472	0.02106	0.00501	0.283769	0.697665

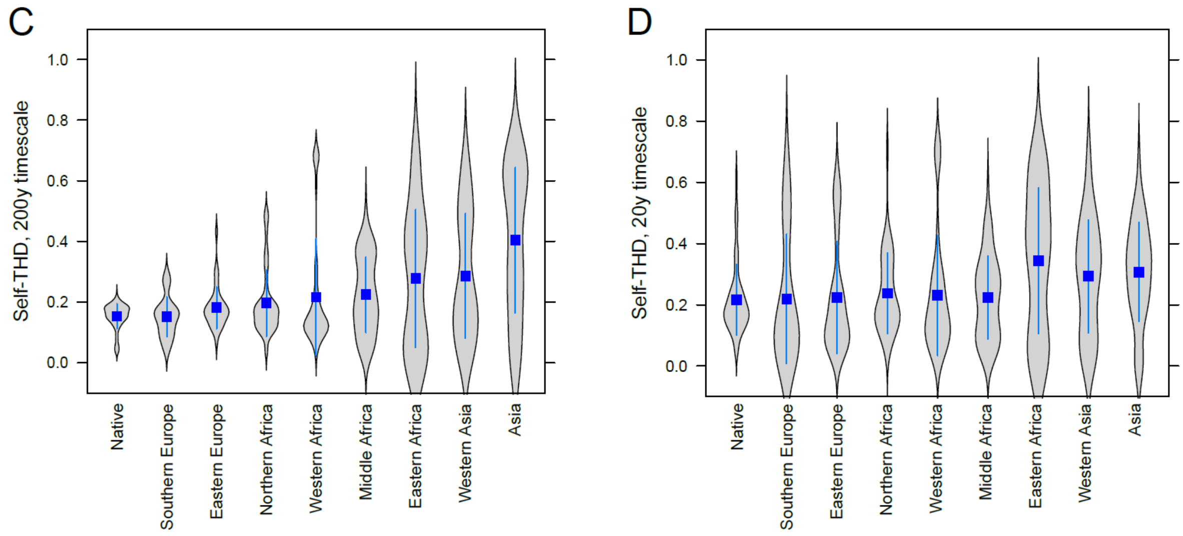

Supplementary Figure 1. Distribution of self-THDs in isolates from French-native and non-native patients with tuberculosis.

Shown are density (violin) plots of the distribution of self-THDs grouped by the region of origin of each patient, using a long- or a short-term timescale (C and D, respectively). Blue squares and lines denote mean and standard deviation. P-values of pairwise comparisons between groups are shown in A (long-term timescale) and B (short-term timescale). Significant comparisons are highlighted. Comparisons were based on Welch's *t*-test without correction for multiple testing. Sample sizes in each group are indicated in Supplementary Table 1.

Supplementary Methods 1. Practical implementation of THD and cross-THD analyses.

Source code of THD routines for the R platform are provided in the Supplementary Text file. Here we provide a brief example of practical THD computation with R commands.

Input data format. THD computations rely on Hamming genetic distances, defined as the number of differences between individuals. The routines expect as input a matrix where rows are individuals and columns are marker values. These values can be of any type as long as they can be compared, including nucleotides, amino-acid codes, or minisatellite repeat numbers.

Parameters. Model parameters include the timescale, the per-marker mutation rate and the number of markers. The timescale defines the length of evolution time before present considered in the THD model. Its unit must be consistent with that used for the mutation rate. The mutation rate is the probability of change per marker and per unit of time. The number of markers is the maximal number of differences between two individuals. When using minisatellite data, this number equal the number of sites, hence the number of columns in the data matrix. When using SNP data, this number can be greater than the number of columns of the data matrix when invariant positions have been removed.

Example source code for cross-THD analysis.

Assuming that the THD source code file is named "thd.R", use

```
source("thd.R")
```

to load functions in the R environment. Cross-THD computations are performed by the high-level function `crossthd`.

Suppose the data matrix represents 15-loci minisatellite data for 100 individuals that evolved for 4 isolated clades of 25 individuals each. We can simulate such a matrix with Poisson random draws of the number of repeats at each position.

```
p <- 15
groupsize <- 25
data <- rbind(
      matrix(rpois(groupsize*p, .1), groupsize, p),
      matrix(rpois(groupsize*p, .2), groupsize, p) + 1,
      matrix(rpois(groupsize*p, .4), groupsize, p) + 2,
      matrix(rpois(groupsize*p, .8), groupsize, p) + 3
)
```

Suppose the group of each isolate is stored in a `groups` factor.

```
groups <- factor(LETTERS[rep(1:4, each = groupsize)])
```

We simulate movements of individuals between groups by randomizing one-fourth of the group labels.

```
groups[sample(1:length(groups))[1:groupsize]] <- sample(
      levels(groups), groupsize, replace = T
)
```

The four clades can be visualized on a dendrogram,

```
hc <- hclust(as.dist(hamming(data)), method = "average")
plot(hc, labels = groups, xlab = "", main = "", sub = "", hang = -1, cex =
0.5)
```

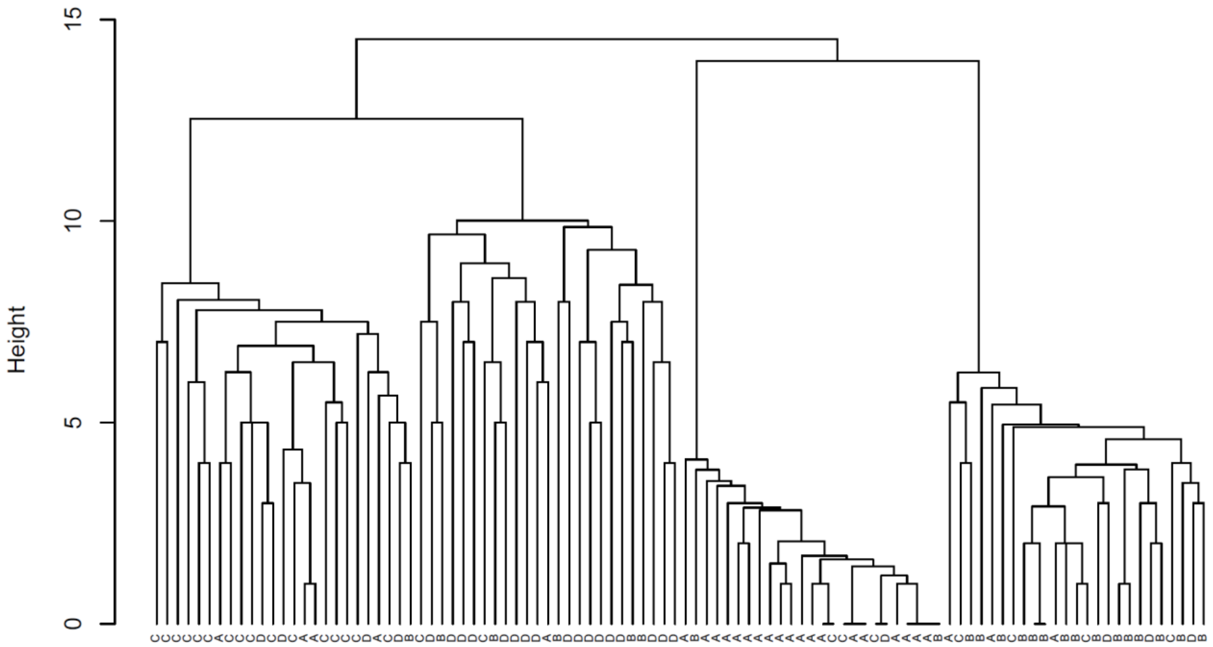

Finally, we compute the cross-THDs between the groups using the crossthd function. Here we use a mutation rate of 5.10^{-4} change per marker per year and a timescale of 50y.

```
crossthd(data, groups, timescale = 50, mu = 5e-4, simplify = T)
```

	A	B	C	D
A	0.70	0.08	0.05	0.20
B	0.09	0.66	0.13	0.10
C	0.05	0.10	0.74	0.10
D	0.16	0.14	0.13	0.60
